# Application of the Microwave Technique in Continuous Flow Processing of Organophosphorus Chemical Reactions

**DOI:** 10.3390/ma12050788

**Published:** 2019-03-07

**Authors:** Erika Bálint, Ádám Tajti, György Keglevich

**Affiliations:** Department of Organic Chemistry and Technology, Budapest University of Technology and Economics, 1521 Budapest, Hungary; ebalint@mail.bme.hu (E.B.); tajti.adam@mail.bme.hu (A.T.)

**Keywords:** microwave irradiation, microwave reactors, continuous flow technique, organophosphorus syntheses

## Abstract

The microwave (MW) technique is an efficient tool in the realization of organic reactions, as well as in the analytical field and in the food industry. The continuous flow approach is of special interest as a promising way to scale-up MW-assisted syntheses. Besides summarizing the batch precedents, this review focuses on the utilization of the MW technique in the continuous-flow realization of organophosphorus transformations. The advantages of the continuous flow technique against the batch accomplishment are also shown. A few materials chemistry-related applications are also mentioned.

## 1. Introduction

### 1.1. Batch vs. Continuous Flow Systems

Continuous procedures have been playing a significant role in important fields like the oil, plastic, and fine chemical industries, or metal processing for a long time [1]. The paradigm shift from batch reactions to continuous accomplishments is the consequence of progress in organic chemistry, and came to the front in the last decade [2].

**Figure 1:**
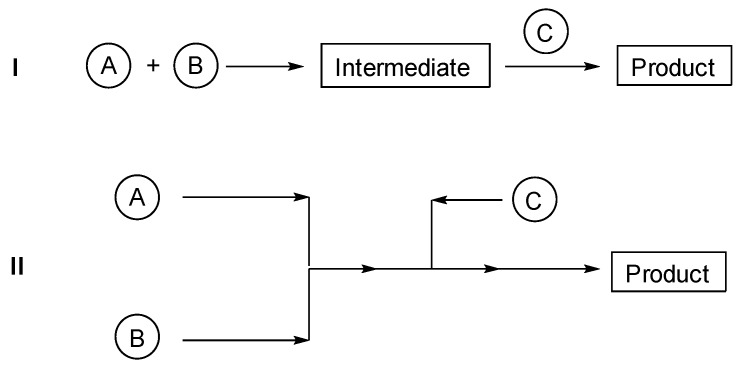
Schematic drawings of a batch and a continuous flow system.

As compared to a step-by-step batch reaction (Figure 1/I), where the most important parameters are the concentration of the starting materials, the temperature, and the reaction time, in continuous flow operations (Figure 1/II) the outcome can be controlled by the flow rate of the reagents and the average residence time set in the reactor. A continuous flow reaction can reach a steady state when the intake and the output are stationary. This means that the product is obtainable continuously in a permanent quality [3].

Flow reactors offer a basically different processing environment [4]. In a typical flow reactor, relatively small quantities are present in the reacting volume, allowing for a better mixing and a faster response on parameter adjustments. The precise parameter control is the key factor to obtain the target molecules with better purity and selectivity, as well as in higher yields. Continuous processes also mean an improvement regarding safety, as the handling of toxic or unstable intermediates becomes easier when smaller amounts are used [5]. Due to the low volume ratio and small quantities, the temperature regulation is simple, which makes the overall reaction safer. Moreover, flow reactors do not contain any gas volume, which enables reaction temperatures exceeding the boiling point of the solvent more easily.

An up-to-date and safe synthesis requires a fully controlled process [6]. Flow reactors can be readily coupled with tools of the process analytical technology (PAT), allowing real-time monitoring and the possibility for automation. Due to the easy monitoring and constant product quality, a continuous flow reactor can be an ideal choice for syntheses in the pharmaceutical industry [7].

The scale-up of flow reactions should be mentioned as a possible drawback [8]. This problem may be overcome by operating a series of parallel flow reactors of the same size. The flow reactions under discussion require a homogeneous medium with low viscosity, which may also mean a challenge in certain applications. The price of the necessary equipment for a continuous process is usually higher compared to that of a batch reactor [9].

### 1.2. Batch Microwave Chemistry

Microwave (MW) irradiation is widely used as a heat source in different fields, such as organic, inorganic, and analytical chemistry, finding applications in the medicinal, polymer, and food industries, as well as in the areas of material processing [10,11].

The evolution of MW-assisted processes started in the 1950s [12]. For three decades, the MW technique was mainly used in the food industry or as a heat source for drying. The first chemistry-related applications, e.g., sample preparation and digestion in analytical chemistry, also appeared during this period. The first MW-assisted chemical syntheses were introduced in 1986 [13,14]. Since then, thousands of publications have been published in this area.

Early synthetic attempts using MW irradiation were carried out in kitchen MW ovens [12]. In these devices, the MW irradiation is controlled by turning on and off the magnetron, which does not allow continuous irradiation and makes the process uncontrollable. The lack of precise measurement and control of the most important parameters (temperature and pressure) makes these experiments unreproducible. The problem was recognized, and this initiated the development of professional MW reactors designed for laboratory use. The first commercially available MW reactors appeared on the market in the early 2000s. The improvement from household MW ovens was significant. These MW reactors were equipped with a specially designed MW cavity, a built-in magnetic stirrer, and a precise temperature sensor, and also offered a connection to a PC for the monitoring and control of the experiments. The use of professional MW chemistry became broadly available, and this multiplied the amount of publications in the area under discussion.

In contrast to conventional heat sources, MW can directly heat the molecules in the bulk of a mixture [15]. Besides the energy efficiency, MW heating has several other benefits, such as accelerating reactions and offering milder reaction conditions, higher yields, and better selectivities, as well as allowing solvent- and/or catalyst-free methods.

As the mechanism of MW heating requires the interaction of MWs and the material, the dielectric constants of the reagents have to be checked before designing a MW-assisted synthesis [10]. At least one of the starting materials should be polar enough to be able to absorb the MW energy. In certain cases, polar additives (e.g., polar catalysts, salts, or ionic liquids) may be added to increase the polarity of the mixture [16,17,18].

Over the years, the MW technique has proven to be efficient in a wide variety of syntheses. Among these, a few reaction types like couplings, multicomponent reactions, condensations, and cycloadditions turned out to be especially advantageous under MW conditions [19].

Although the MW technique has several benefits, the scale-up suffers from a serious limitation [12]. The MW irradiation is generated by a magnetron coil, which has a defined geometry. Structural materials, like borosilicate glass or Teflon, are needed for MW equipment requiring a special design, not allowing for the establishment of larger-scale reactors. Manufacturers of MW devices offer multimode reactors, where 6–12 samples can be heated parallelly. However, this is far away from a real scale-up, which is inevitable for industrial-like applications. A more suitable approach allowing MW-assisted syntheses in larger quantities is discussed in the next chapter.

### 1.3. Continuous Flow Microwave Attempts

According to a review in the field of MW-assisted material chemistry [20], continuous processes have been developed for the preparation of carbides, sintering of ceramics, or the continuous treatment of oil-contaminated drill cuttings. These instances differ from the usual flow reactions discussed in this subchapter, as the feed of the reactor is not a homogenous solution.

In MW-assisted organic chemistry, the continuous flow technique is of special interest. Combining MW heating with the continuous flow technique creates a promising way to scale-up MW-assisted syntheses [12,15,21,22,23]. The first systems coupling MWs and continuous flow were applied to polymer heating and solid drying in the 1980s [24,25]. The continuous flow MW reactors for chemical reactions appeared in the 1990s [26]. Since then, the number of publications in this field has been constantly increasing, and until the present more than 300 papers have been published. The advantages of continuous flow MW reactors were reported for a series of transformations. However, in many cases, fabricated domestic MW ovens were used. Due to the uncontrolled temperature and pressure in these non-professional MW “reactors”, it is practically impossible to reproduce these transformations [23,27]. Gradually, professional MW-flow systems with accurate temperature measuring devices were also developed [28,29].

**Figure 2:**
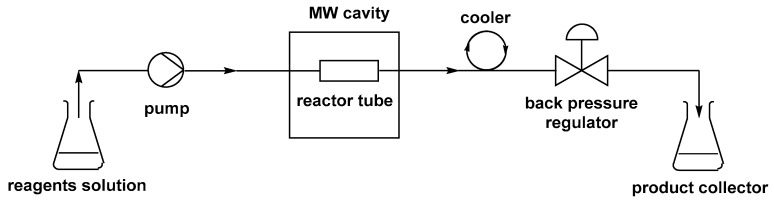
Schematic drawing of continuous flow microwave (MW) systems.

The continuous flow MW systems usually consist of three main parts: the dispensing units for the starting reagents, the MW cavity, and the product collector (Figure 2). The reagents are fed into the reactor using one or more pumps that may be HPLC or syringe pumps. The pressure is usually controlled by a back-pressure regulator while the temperature is monitored using a built-in IR sensor or a fiber optic sensor. In most cases, the flow reactor is made of MW transparent Pyrex or Teflon. Further scale-up of flow MW systems can be realized by applying parallel reactors.

There are many types of continuous flow MW reactors [21], which contain a normal flask or tube [30], an Ω- or U-shaped tube [31,32], a spiral glass tube [30,33,34,35], or a mixed tube [36] to accommodate the reactive volume. Furthermore, reactor designs of fixed bed tubular coils [37,38], filled columns [37,38,39], and capillary reactors [40,41,42] are also available. Coupling MW heating and microreactors is also a challenging endeavour [23].

There are several papers, which reported on continuous flow MW accomplishments in the g or kg scales [27,43,44,45,46,47,48,49,50,51,52,53,54,55,56,57], or even larger scales (500 kg product per day) [58].

The spread of MW and continuous flow techniques have resulted in an enormous development in synthetic chemistry. Continuous flow MW reactors make possible efficient, fast, and selective transformations, as well as precise monitoring and control of the reaction parameters.

### 1.4. Microwaves in Batch Organophosphorus Syntheses

Guenin summarized the early results obtained in MW-promoted P-chemistry [55].

**Scheme 1:**
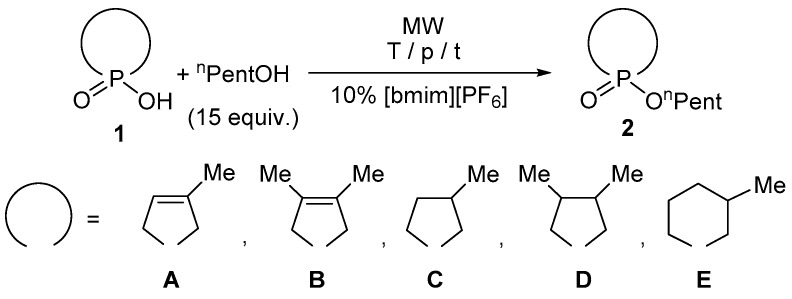
Direct esterification of ring phosphinic acids (**1)** by pentanol using [bmim][PF_6_] as the catalyst.

It was found by us that P-hydroxy phospholene oxides, -phospholane oxides, and -hexahydrophosphinine oxides (**1**) could be esterified with alcohols under MW conditions to provide the respective phosphinates (**2**) in yields of 31–82% [56,57,58,59,60]. Although this protocol means a green way to prepare the phosphinates, the temperatures of 200–235 °C and the times of up to 5 h are disadvantageous. Later on, we observed that, in the presence of 10% of ionic liquids (ILs), the esterifications were more efficient. This was shown via the esterification of different ring phosphinic acids (**1**) with pentanol (Scheme 1). Among the imidazoliaun salts tested as catalysts, [bmim][PF_6_] was found to be the most appropriate [61]. The use of this additive made almost complete conversions possible, as well as higher yields (72–94%) at lower temperatures of 180–220 °C after shorter reaction times of 0.5–2 h. The positive effect of the IL additives is the consequence of their better MW absorbing ability.

**Scheme 2:**
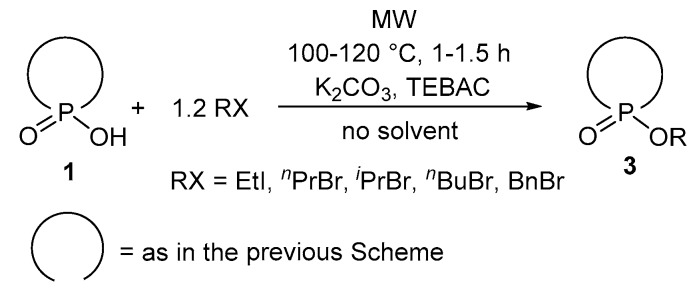
The reaction of ring phosphinic acids (**1**) with alkyl halides.

The solid–liquid heterogeneous phase alkylation of ring phosphinic acids (**1**) was studied by alkyl halides under MW irradiation, using K_2_CO_3_ to remove the liberated hydrogen halogenide (Scheme 2) [57,62]. The effect of triethylbenzylammonium chloride (TEBAC) as a phase transfer catalyst (PTC) was also investigated. In the cases of the alkylation of 1-hydroxy-3-phospholene oxides (**1A** or **1B**), it was found that by using benzyl bromide, which is of increased reactivity, the respective phosphinates (**3A** and **3B**) were obtained in yields of 92%/84% (also in the absence of a catalyst). At the same time, when alkyl halides of normal (*^n^*Pr- and *^n^*Bu-bromide) or decreased reactivities (*^i^*Pr-bromide) were applied, the presence of TEBAC enhanced the reactions. Yields of 90–96% were obtained for derivatives with normal alkyl chains. In these cases, the effect of the catalyst was synergetic with the MWs. The alkylations were then extended to the esterification of 1-hydroxyphospholane oxides (**1C** and **1D**) and a 1-hydroxy-hexahydrophosphinine oxide (**1E**). The respective esters were obtained in rather good yields (81–98%).

**Scheme 3:**
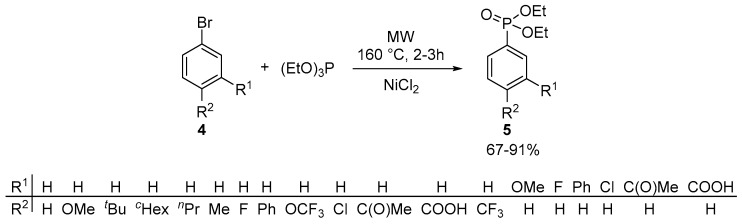
Arbuzov reaction of aryl bromides and triethyl phosphite.

Aryl phosphonates (**5**) may be intermediates for arylphosphonic acids. A MW-promoted, NiCl_2_-catalyzed Arbuzov reaction of aryl bromides (**4**) with triethyl phosphite was developed by our team (Scheme 3). In this case, a catalytic transformation was assisted by MWs [63,64].

**Scheme 4:**
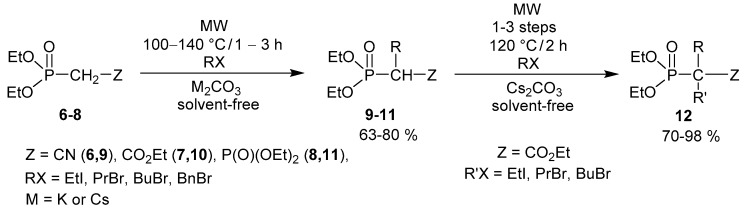
Alkylation of active methylene-containing phosphonates.

The solid–liquid phase alkylation of CH acidic P-derivatives, such as diethyl cyanomethylphosphonate (**6**), diethyl ethoxycarbonylmethylphosphonate (**7**), and tetraethyl methylenebisphosphonate (**8**), providing products **9**–**11**, was developed under MW irradiation (Scheme 4) [65,66,67]. There was no need to apply a PTC under MW irradiation [68,69]. A protocol for the di-substitution of diethyl ethoxycarbonylmethylphosphonate (**7**) was also worked out [70].

**Scheme 5:**
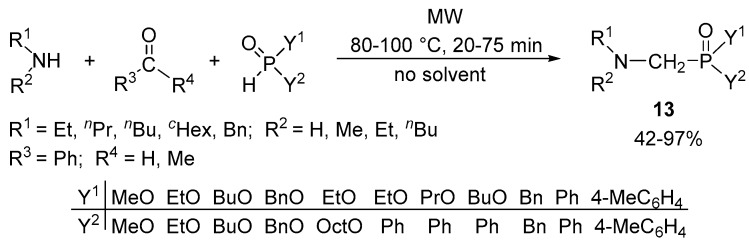
Simple Kabachnik–Fields reaction.

It was found that there was no need for a catalyst in the Kabachnik–Fields reactions carried out under MW conditions [71,72]. A series of α-aminophosphonate derivatives (**13**) was synthesized by the three-component reactions of primary or secondary amines, oxo-compounds, and different >P(O)H reagents, like dialkyl phosphites, alkyl phenyl-*H*-phosphinates, and secondary phosphine oxides (Scheme 5) [71,72,73,74,75,76].

**Scheme 6:**
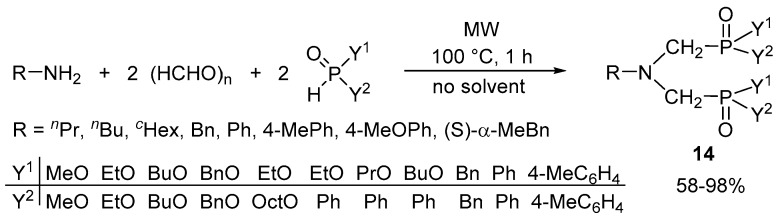
Double Kabachnik–Fields reaction starting from primary amines.

Double Kabachnik–Fields reactions were also developed, where the primary amines were reacted with two equivalents of paraformaldehyde and the same amounts of the >P(O)H species under MW irradiation in a catalyst-free manner (Scheme 6) [73,74,75,76,77,78,79]. The respective products (**14**) could be prepared in variable yields. After double deoxygenation of the bis(aminophosphine oxides) (**14**, Y^1^ = Y^2^ = Bn, Ph or 4-MeC_6_H_4_), the bisphosphines obtained were used as bidentate P-ligands in the preparation of ring Pt complexes [76,77,78,79].

**Scheme 7:**
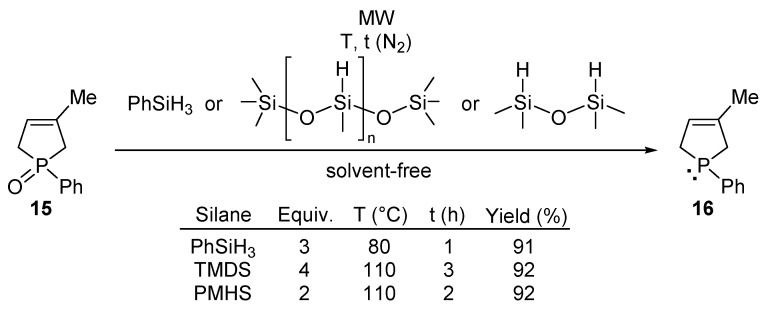
Reduction of a 3-phospholene oxide by cheap silanes.

The deoxygenation of phosphine oxide by-products is challenging. Cl_3_SiH or PhSiH_3_ are suitable reducing agents, however, the previous reagent is corrosive, while the latter one is expensive. In replacement of these reagents, the cheap (Me_2_SiH)_2_O (TMDS) and polymethylsiloxane (–(OSiMeH)_n_–; PMHS) were used under MW conditions. It was proved that under MW irradiation, there was no need for any catalyst proposed by Beller et al. [80]. This is exemplified by the reduction of 1-phenyl-3-methyl-3-phospholene 1-oxide (**15**) using PhSiH_3_, TMDS and PMHS, as summarized in Scheme 7 [81,82,83,84].

**Scheme 8:**
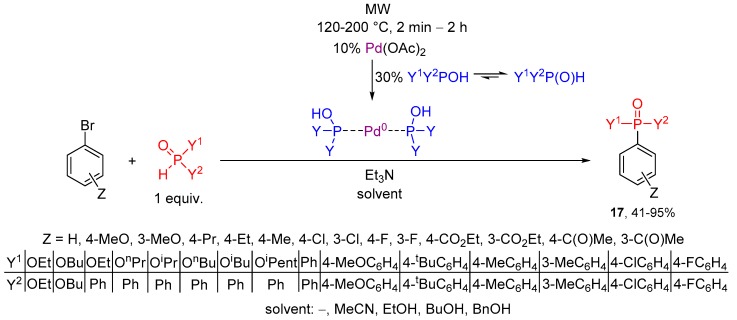
The P–C coupling reaction of bromobenzenes and >P(O)H reagents.

The first P–C coupling reaction between aryl- or vinyl-halides and dialkyl phosphites furnishing phosphonates was described by Hirao using Pd(PPh_3_)_4_ as the catalyst [85,86]. Later on, other methods were also reported to replace the Pd(0) catalyst with Pd(II) or another metal (e.g., Ni(II) salts applied together with mono- or bidentate P-ligands). The P–C coupling was then extended by utilizing other >P(O)H reagents, bases, and solvents. Our research group found that the P–C coupling of aryl-bromides and different >P(O)H species, like dialkyl phosphites, ethyl phenyl-*H*-phosphinate, and diphenylphosphine oxide may take place in the presence of Pd(OAc)_2_ as the catalyst precursor under MW conditions without the addition of the usual P-ligands, but using the P-reagent in some excess (Scheme 8) [87,88]. The ArP(O)< products (**17**) were obtained in variable yields. Our idea was that the excess of the >P(O)H compounds existing under a tautomeric equilibrium (with the >P-OH form) may act as P-ligands [89].

## 2. Microwave-Assisted Continuous Flow Applications

### 2.1. Development of the Continuous Flow Microwave Device

Among continuous flow MW-assisted syntheses, there are examples for esterifications [27,44,46], acylations [27,44,46], multicomponent [27] and rearrangement reactions [27,28,44], couplings [27,28,43,44], and polycondensations [90]. The simplest model is the esterification of carboxylic acids with alcohols, where different types of flow cells (e.g., teflon coils, glass tubes, or ceramic tubes) were applied in kitchen MW ovens [91,92] or in professional MW reactors [93,94].

**Figure 3:**
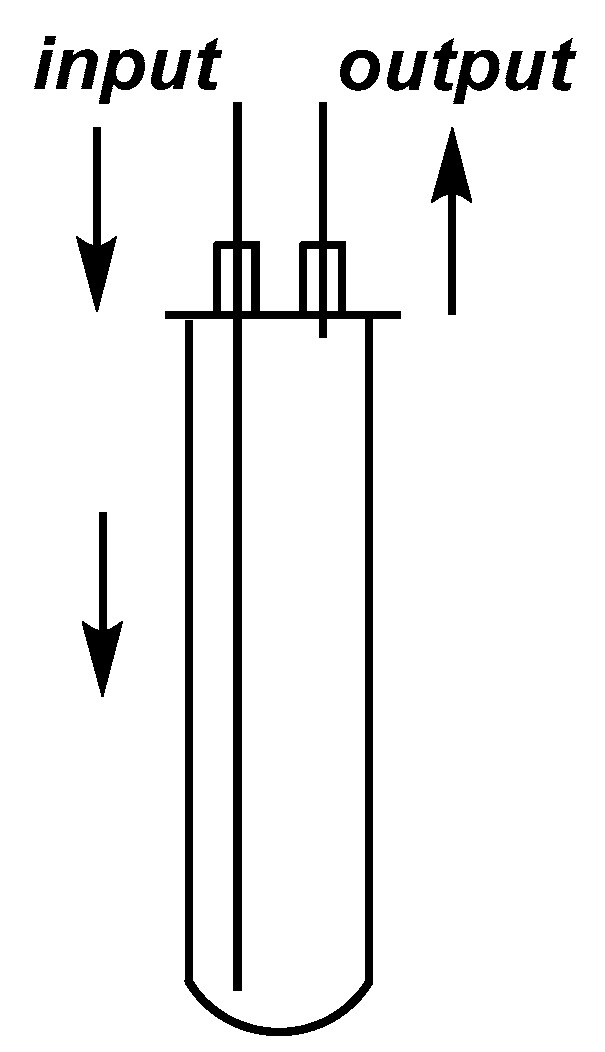
Sketch of the continuous flow cell.

A continuous flow MW system (Figure 2, Section 1.3) was developed by us, using a commercially avialable flow cell (Figure 3) immersed into a CEM^®^ MW reactor [95]. The mixture of the reagents is fed into the reactor by a HPLC pump. The temperature of the mixture is monitored and controlled by an IR sensor built into the reactor. After irradiation at the desired temperature, the leaving reaction mixture is cooled down in a spiral-like cooler, and is passed through a back pressure regulator operating at 250 PSI.

The first model reaction to be investigated by us in the flow system developed was the direct esterification of benzoic acid with various aliphatic alcohols [95]. At first, batch reactions were carried out to investigate the reaction. The reactivity of the alcohols towards benzoic acid was mapped and the parameters were optimized. Based on the batch results, the esterification rate of benzoic acid depends on the boiling point (from EtOH to *^n^*PentOH) and the steric properties of the alcohols (*^n^*Pr vs *^i^*Pr). Then, the esterifications were optimized further in the continuous flow MW reactor. It was found that when using 15 equivalents of alcohol at 140 °C at a flow rate of 0.35–0.25 mL/min (with a residence time of 20–30 min), the direct esterifications were complete and the corresponding alkyl benzoates were obtained in yields of 95–98%.

### 2.2. Elaboration of the Continuous Flow Transesterification of Dialkyl Phosphites

**Scheme 9:**
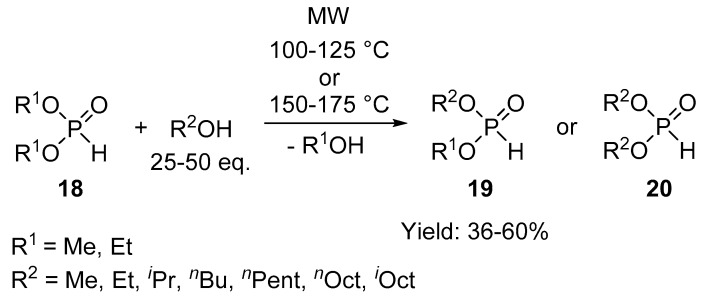
Batch alcoholysis of dialkyl phosphites by simple alcohols.

The MW-assisted transesterification of dialkyl phosphites (dialkyl *H*-phosphonates) was studied with simple alcohols (Scheme 9) under catalyst-free conditions [96]. These compounds may serve as starting materials, for example in the Kabachnik–Fields condensations or in the aza-Pudovik reactions, to provide potentially bioactive α-aminophosphonates [97]. As the first step, the transesterifications were performed under batch conditions. It was found that, depending on the conditions (molar ratio, temperature, and reaction time), the alcoholysis of dialkyl phosphites (**18**) resulted in the formation of dialkyl phosphites with two different (**19**) or with two identical (**20**) alkyl groups in different ratios. Dialkyl phosphites with two different alkyl groups (**19**) are valuable building-blocks in the synthesis of chiral organophosphorus derivatives [73].

**Scheme 10:**
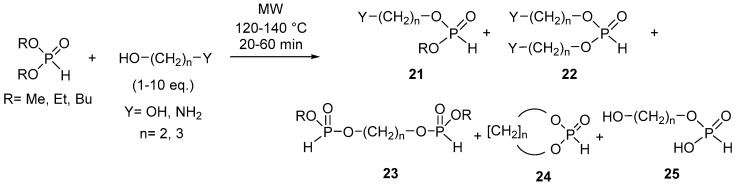
Batch alcoholysis of dialkyl phosphites by diols and aminoalcohols.

Hydroxyalkyl- or aminoalkyl-functionalized *H*-phosphonates could be synthesized by the alcoholysis of dialkyl phosphites with glycols or amino alcohols (Scheme 10) [98,99]. During the investigation of this reaction, it was found that mixed phosphites (**21**) and/or fully transesterified products (**22**) were formed as major components, depending on the ratio of the starting materials, the temperature, and the reaction time. These derivatives may be starting materials in the synthesis of P-containing polymers, which form a special group within plastics [100,101]. In certain cases, a small amount of by-products (**23**–**25**) was also present in the reaction.

**Scheme 11:**
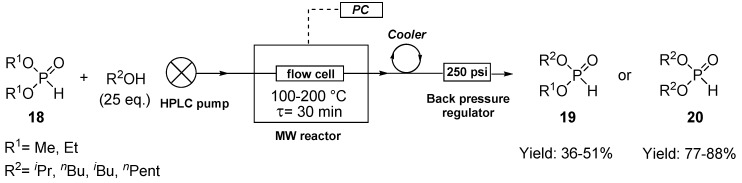
Continuous flow MW system for the alcoholysis of dialkyl phosphites.

The continuous flow alcoholysis of dialkyl phosphites by aliphatic alcohols in the absence of catalyst was also elaborated using the same continuous flow MW system applied in the esterification of benzoic acid (Scheme 11) [102]. By the precise control of the reaction parameters, the alcoholysis could be fine-tuned towards dialkyl phosphites with two different (**19**) or with two identical alkyl groups (**20**). Although the selectivity of the reaction was rather similar under flow and batch conditions, in the continuous flow reactor, the dialkyl phosphites with two different alkyl groups were obtained at a shorter reaction time in a higher proportion with a somewhat higher productivity.

### 2.3. Continuous Flow Synthesis of α-Aminophosphonates by the aza-Pudovik Reaction

**Scheme 12:**
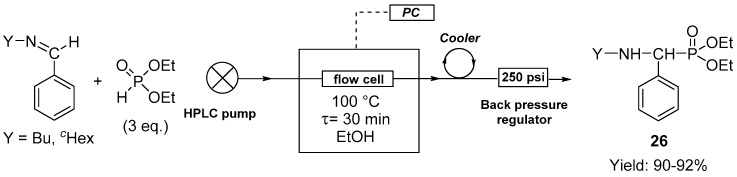
Continuous flow synthesis of α-aminophosphonates by the aza-Pudovik reaction.

We elaborated the MW-assisted continuous flow synthesis of potentially biologically active α-aminophosphonates by the aza-Pudovik reaction, involving the addition of dialkyl phosphites to the C=N double bond of imines (Scheme 12) [103]. Before optimization, we carried out the flow-compatible addition based on our previous experiences [104]. In this simple two-component model reaction, the reagents were fed together into the flow cell. The MW-assisted catalyst-free continuous flow reaction of *N*-benzylidene-butylamine or *N*-benzylidene-cyclohexylamine and diethyl phosphite in ethanol afforded the corresponding α-aminophosphonates (**26**) in yields of 90–92%.

### 2.4. The Synthesis of α-Aminophosphonates by Continuous Flow Kabachnik–Fields Reaction

**Scheme 13:**
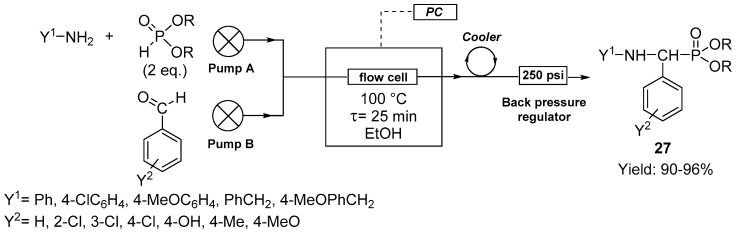
Continuous flow synthesis of α-aminophosphonates by the Kabachnik–Fields reaction.

An efficient synthetic route towards α-aryl-α-aminophosphonates is the three-component Kabachnik–Fields reaction of primary amines, benzaldehyde derivatives, and dialkyl phosphites. The continuous flow variation was elaborated by us (Scheme 13) [103]. To avoid the possible side reactions of the starting materials at room temperature resulting in the formation of an imine or an α-hydroxyphosphonate, a dual-pump continuous flow system had to be developed. The mixtures of the primary amine and diethyl phosphite in ethanol (pump A), and benzaldehyde in ethanol (pump B) were fed separately into the mixer of the HPLC pump. After optimization of the conditions, the continuous flow method was proved to be more efficient than the batch approach, as the corresponding α-aminophosphonates (**27**) were obtained in a shorter reaction time, and a lower excess of phosphite was needed.

## 3. Conclusions

The recent paradigm shift from batch processes to continuous flow operations has brought about a significant change in organic chemistry, and meant a challenge in organophosphorus chemistry. The continuous flow technique means improvements in respect to safety, selectivity, and efficiency, and makes possible the development of automated syntheses more easily. MW chemistry has come to the front in the 1980s. Since then, the MW technique has spread in various fields, including organic, analytical, and material chemistry. Many types of reactions were proved to be more efficient under MW conditions. In the field of organophosphorus chemistry, several transformations, such as the esterification of phosphinic acids, O- and C-alkylations, Kabachnik–Fields condensation, the reduction of phosphine oxides, and P–C couplings all take advantage of MW irradiation. By combining flow chemistry and the MW technique, besides the benefits mentioned above, the easy scale-up of MW-assisted reactions has also become possible. The elaboration of the alcoholysis of dialkyl phosphites and the synthesis of α-aminophosphonates by aza-Pudovik reactions, as well as Kabachnik–Fields reactions in a continuous flow MW reactor, allowed the preparation of the target organophosphorus products more efficiently, meaning an improved productivity as compared to the batch approaches.
